# Supplementary material for: Molecular basis of human trace amine-associated receptor 1 activation
Source: Nat Commun. 2024 Jan 2;15:108. doi: 10.1038/s41467-023-44601-4 (PMC10762035; doi:10.1038/s41467-023-44601-4)
Supplement: Supplementary file 1 — Supplementary Information [file 41467_2023_44601_MOESM1_ESM.pdf]

Supplementary Information for

Molecular Basis of Human Trace Amine-Associated Receptor 1 Activation

Gregory Zilberg*, Alexandra K. Parpounas, Audrey L. Warren, Shifan Yang, Daniel Wacker*

*To whom correspondence should be addressed: Gregory Zilberg (greg.zilberg@icahn.mssm.edu), Daniel Wacker (Daniel.wacker@mssm.edu)

This file includes:

Supplementary Figures 1-7

Supplementary Tables 1-3

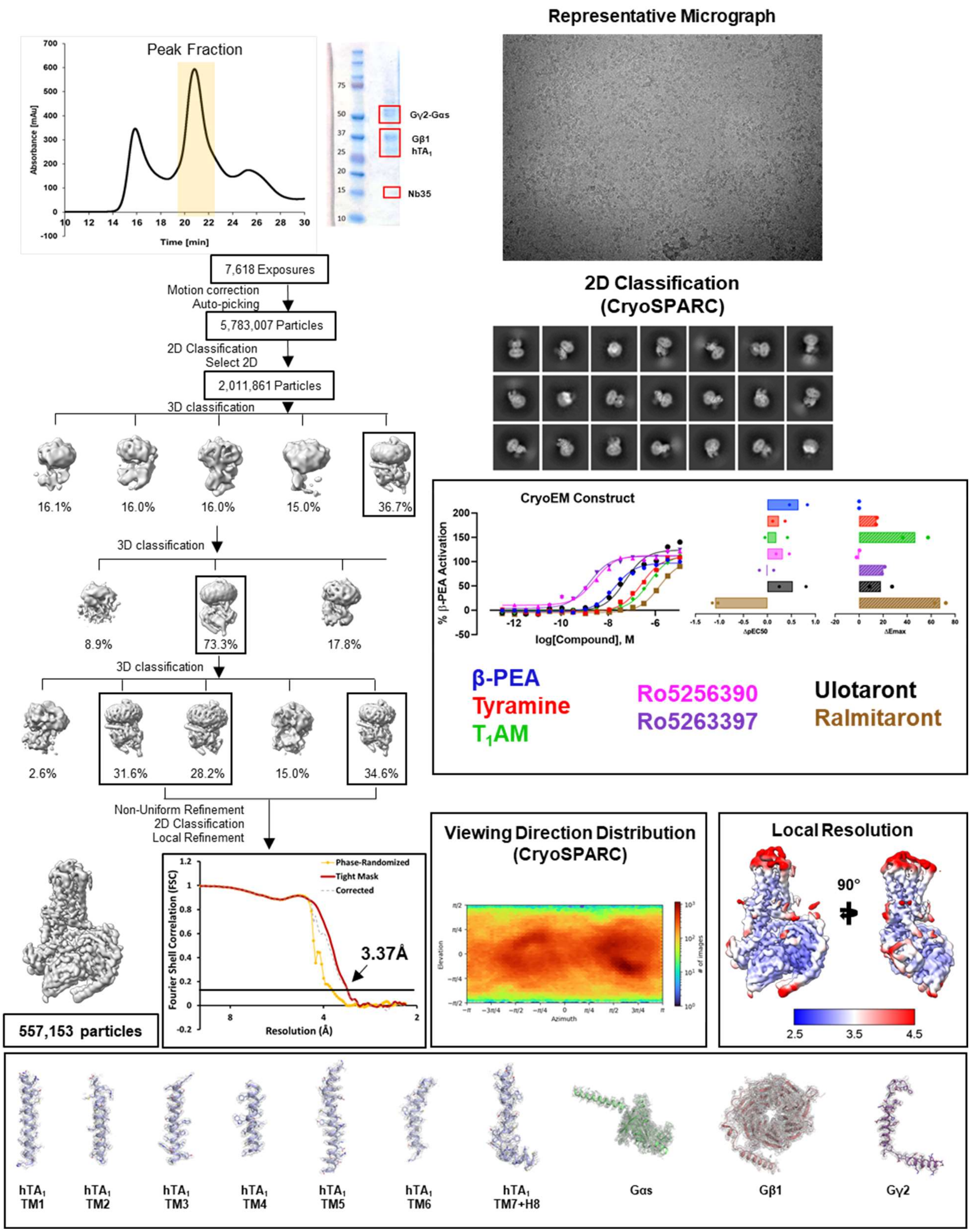

Supplementary Fig. 1 | Purification, and cryo-EM structure determination of the Ro5256390-bound hTA1-Gs-Nb35 complex. Analytical size exclusion chromatography and SDS-PAGE show monodisperse and pure hTA1-Gs-Nb35

complex. Data were collected on a 300 keV Krios, a representative micrograph is shown, and processed in cryoSPARC v4.1.2.: Particles were picked from motion corrected micrographs, subjected to 2D classification (representative classes are shown), followed by ab initio model building and 3D classification. After multiple rounds of 3D classification, the particle stack was subjected to non-uniform refinement. We further removed bad particles through a final 2D classification step, before performing local refinement using a receptor-only mask built based on the mask generated by cryoSPARC for the non-uniform refinement job. A final map was obtained with GS-FSC indicating a resolution of 3.37 Å applying the 0.143 cutoff. Viewing direction distribution analysis (cryoSPARC) indicates sufficient coverage. An initial model was built in PHENIX, and then further refined in ServalCat for the generation of final maps and coordinates. Calculations in cryoSPARC indicate local resolutions of up to 3 Å around the compound binding site. Viewing direction analysis indicates isotropic distribution of views in final particle stack. Bottom panel shows representative cryoEM densities (grey mesh) of receptor helices (light blue), Gαs (green), Gβ1 (salmon), and Gγ2 (purple) subunits at 4σ. Insert also shows activation of the cryoEM construct β_2AR-Nterm-hTA1-F112$^{3.41}$W in transfected HEK293T cells mediated by a panel of agonists. Data represent mean ± SEM of two independent experiments (n=2) performed in triplicate. See Supplementary Table 1 for fitted parameter values. Differences in efficacies and potencies compared to the wildtype construct are shown as bar graphs.

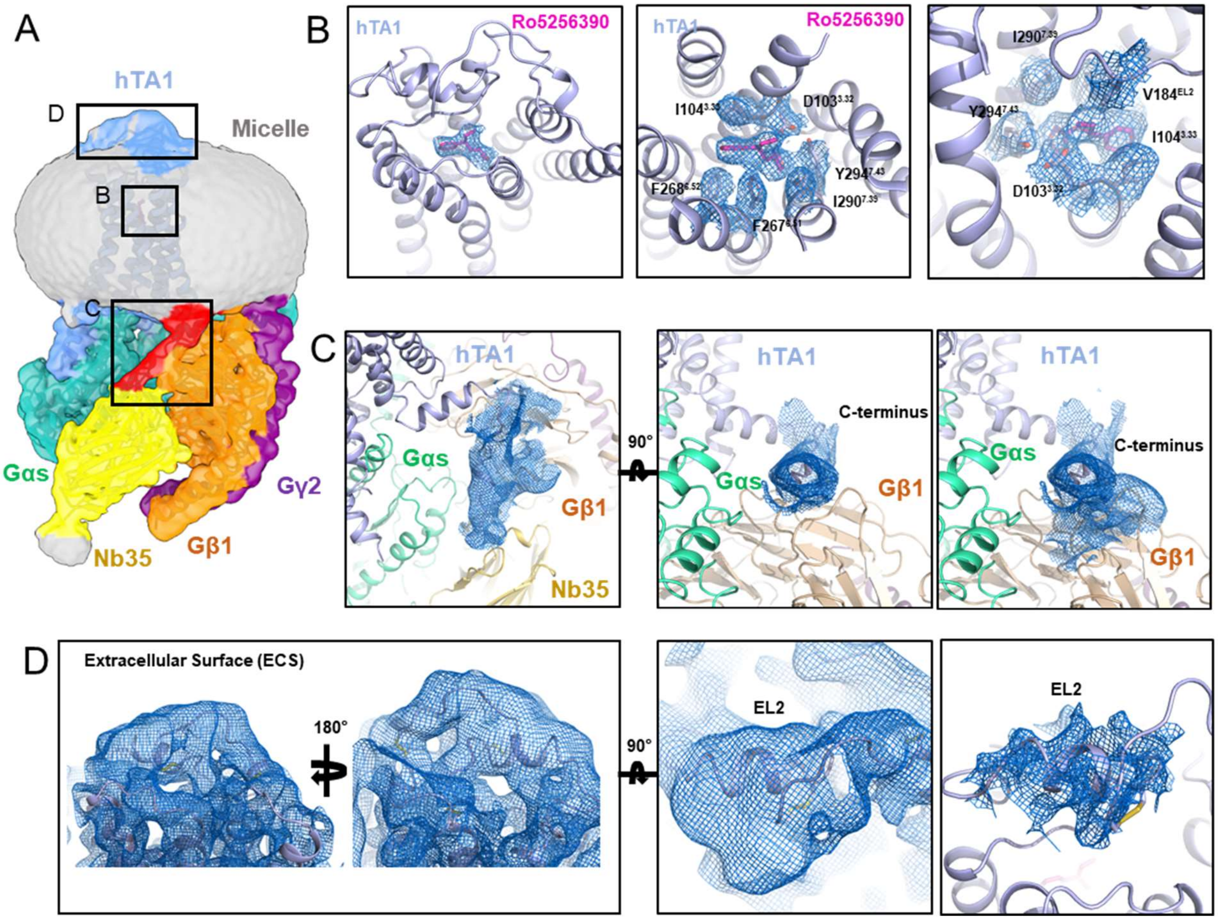

Supplementary Fig. 2 | Representative Cryo-EM density of Ro5256390-bound hTA1-Gs-Nb35 complex. A, Overall unsharpened cryo-EM density of the Ro5256390-bound hTA1-Gs-Nb35 complex, colored by element and featuring inlays demonstrating the relative location of subsequent local density figures B-D. hTA1, light blue; C-terminus of hTA1, red; Ro5256390, magenta; Gαs, green; Gβ1, orange; Gγ2, purple, Nb35, yellow, detergent micelle, grey. **B**, Sharpened cryo-EM density of the ligand Ro5256390 and nearby residues within the OBP shown as blue mesh at a contour level of 6σ. **C**, Unsharpened cryo-EM density of the C-terminal tail of hTA1 packing against G β1 shown as blue mesh at a contour level of 4σ. **D**, Unsharpened cryo-EM density (3 left panels) and sharpened cryo-EM density (right panel) of the hTA1 extracellular surface shown as blue mesh at a contour level of 6σ and 4σ, respectively.

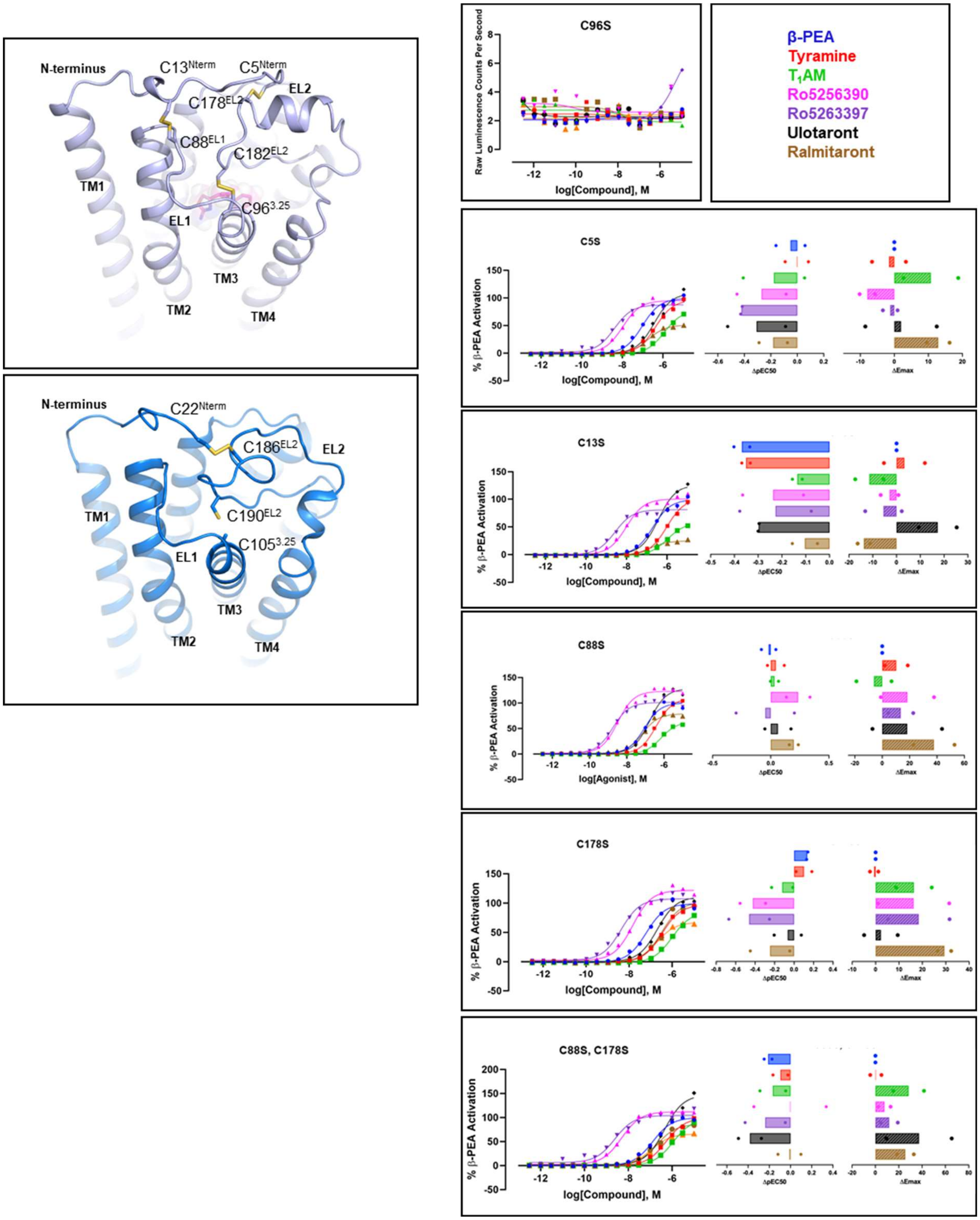
N-terminus
C13^Nterm
C5^Nterm
EL2
C178^EL2
C88^EL1
C182^EL2
TM1
C96^3.25
EL1
TM3
TM2
TM4
N-terminus
C22^Nterm
C186^EL2
EL2
TM1
C190^EL2
C105^3.25
EL1
TM3
TM2
TM4
C96S
Raw Luminescence Counts Per Second
log[Compound], M
β-PEA
Tyramine
T1AM
Ro5256390
Ro5263397
Ulotaront
Ralmitaront
C5S
% β-PEA Activation
log[Compound], M
ΔpEC50
ΔEmax
C13S
% β-PEA Activation
log[Compound], M
ΔpEC50
ΔEmax
C88S
% β-PEA Activation
log[Agonist], M
ΔpEC50
ΔEmax
C178S
% β-PEA Activation
log[Compound], M
ΔpEC50
ΔEmax
C88S, C178S
% β-PEA Activation
log[Compound], M
ΔpEC50
ΔEmax

Supplementary Fig. 3 | Importance of the extracellular disulfide network for activities of hTA1 agonists. Zoom-in on the extracellular disulfide network of hTA1 (light blue) and mTAAR9 (marine blue) with key cysteines shown as sticks (left). Effects of cysteine mutations on the activities of hTA1 agonists (right). Data are shown both as full concentration response curves and as bar graphs denoting differences between mutant and wild type receptor with potencies determined as pEC_{50}s and efficacies measured as E_{max}. Data represent mean ± SEM of two independent experiments (n=2) performed in triplicate. See Supplementary Table 1 for fitted parameter values.

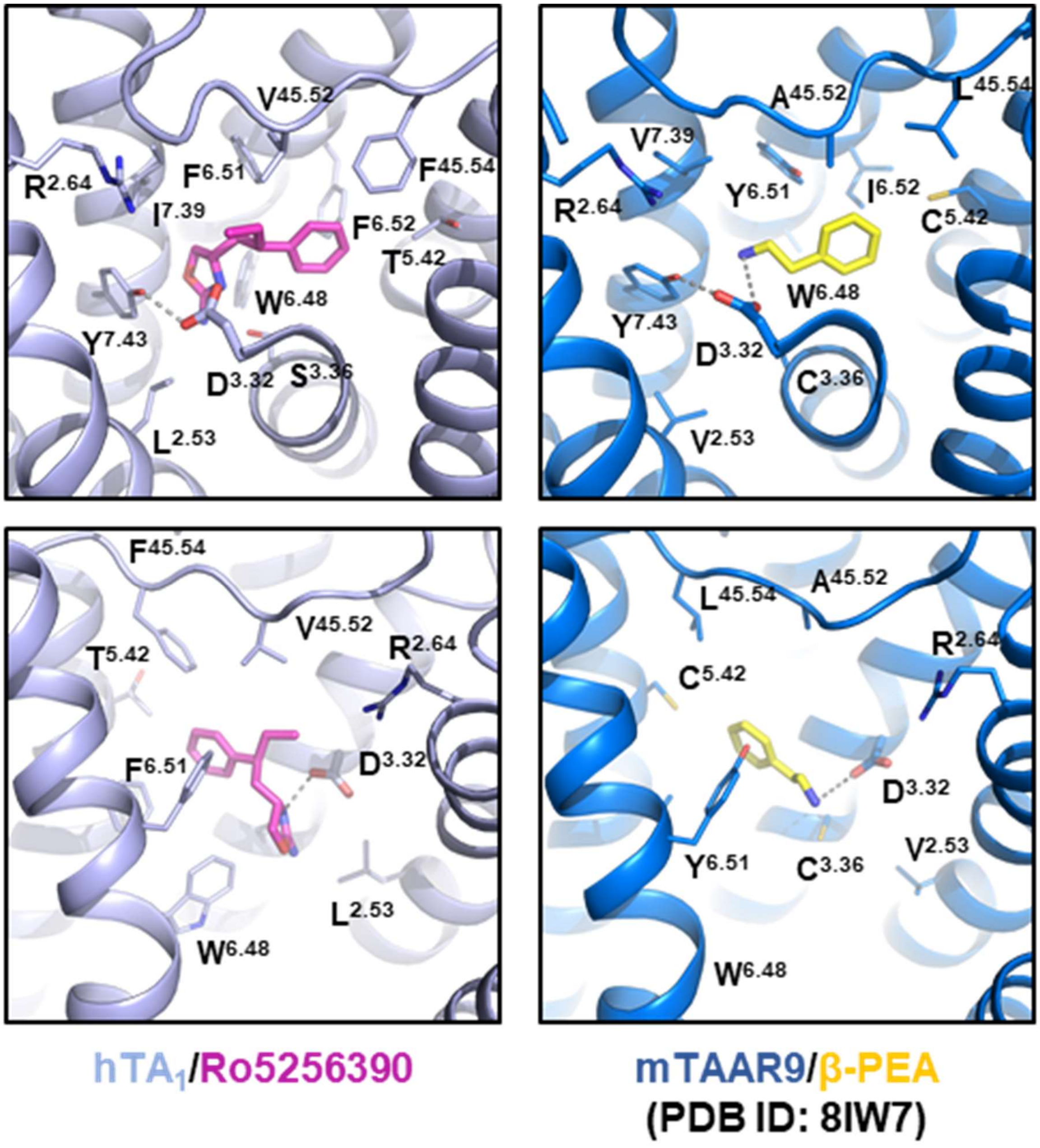

Supplementary Fig. 4 | Role of key OBP residues in the activities of hTA1 agonists. Zoom-in on OBP of hTA1 (light blue) and mTAAR9 (marine blue) with key residues shown as sticks (left). Two angles are shown and grey dashes indicate ionic bonds between the conserved residue D3.32 and the agonists Ro5256390 (magenta) and β-PEA (yellow).

Receptor	Sequence Similarity/Identity [%] (Full Sequence)	Sequence Similarity/Identity [%] (Structurally Conserved Residue Positions)
rTA1	83/77	82/76
mTA1	82/74	86/78

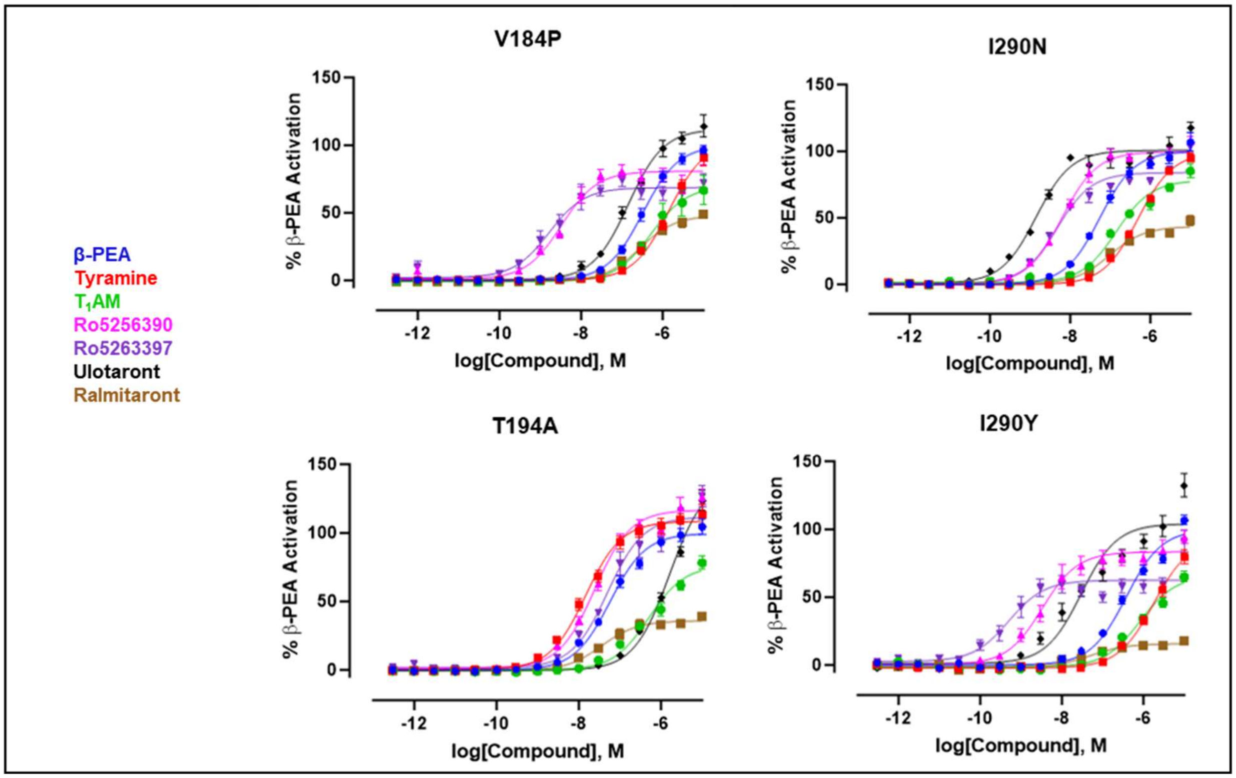

Supplementary Fig. 5 | Implications of species differences for the activities of TA1 agonists. Calculation of sequence similarity and identity for both complete receptor sequences or only structurally conserved positions between human and rodent TA1 (top). Effects of species-related residue substitutions on the activities of TA1 agonists (bottom). Data are shown as full concentration response curves and represent mean ± SEM of two independent experiments (n=2) performed in triplicate. See Supplementary Table 1 for fitted parameter values.

Receptor	Sequence Similarity/Identity [%] (Full Sequence)	Sequence Similarity/Identity [%] (Structurally Conserved Residue Positions)
hTAAR2	56/42	69/53
hTAAR3	60/44	68/50
hTAAR5	51/33	59/41
hTAAR6	54/36	63/43
hTAAR8	49/32	58/39
hTAAR9	54/38	61/43

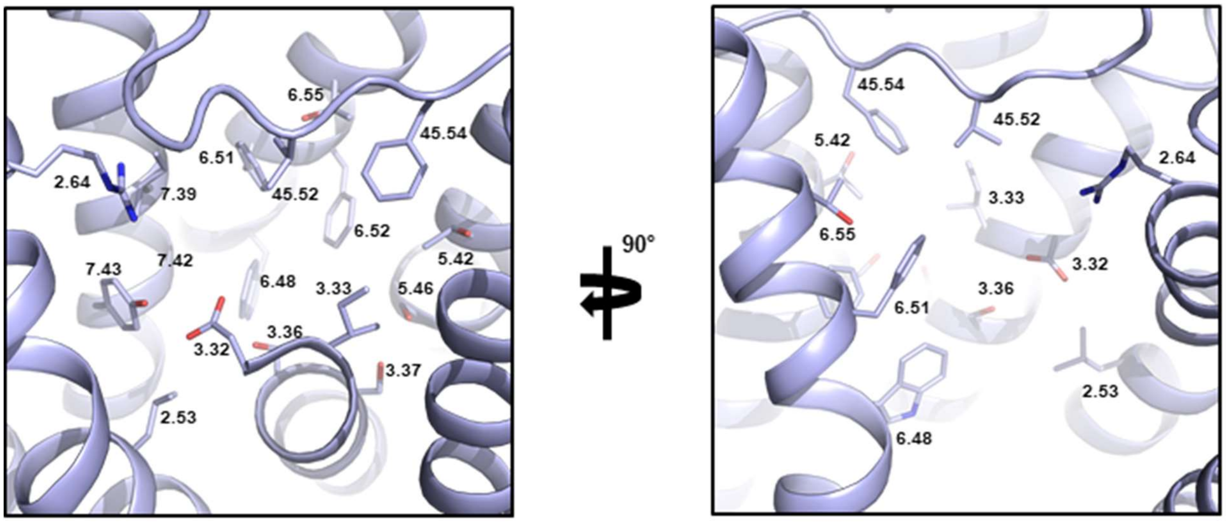

Position	2.53	2.64	3.32	3.33	3.36	3.37	$EL2^{45.52}$	$EL2^{45.54}$	5.42	5.46	6.48	6.51	6.52	6.55	7.39	7.42	7.43
hTA1	L	R	D	I	S	S	V	F	T	S	W	F	F	T	I	G	Y
h5-HT7R	V	T	D	V	C	T	I	Q	S	A	W	F	F	S	L	G	Y
h5-HT2AR	L	T	D	V	S	T	V	D	G	S	W	F	F	N	T	G	Y
h5-HT2BR	V	T	D	V	S	T	L	K	G	A	W	F	F	N	I	A	Y
h5-HT2CR	V	A	D	V	S	T	T	D	G	A	W	F	F	N	C	A	Y
hDRD1	V	A	D	I	S	T	S	L	S	S	W	F	F	N	V	G	W
hDRD5	V	A	D	I	S	T	S	L	S	S	W	F	F	N	V	G	W
h5-HT5AR	V	H	D	V	C	T	L	R	S	A	W	F	F	E	V	R	Y
hTAAR2	L	R	D	L	S	I	V	F	L	G	W	C	F	I	T	G	Y
hTAAR6	V	R	D	V	C	Y	T	V	T	S	W	Y	S	S	C	A	Y
hTAAR5	L	R	D	T	C	L	L	L	L	L	W	F	T	T	I	A	Y

Supplementary Fig. 6 | Similarities in the OBPs of hTA1, TAARs, and aminergic neurotransmitter receptors. Calculation of sequence similarity and identity for both complete receptor sequences or only structurally conserved positions between hTA1

and the other members of the human TAAR family (top). Location of structurally conserved residue positions in the OBP of hTA1 (middle). Sequence alignment of corresponding structurally conserved positions residues (bottom). Most similar sequences are shown and ranked according to analysis done with tools of the GPCRdb.

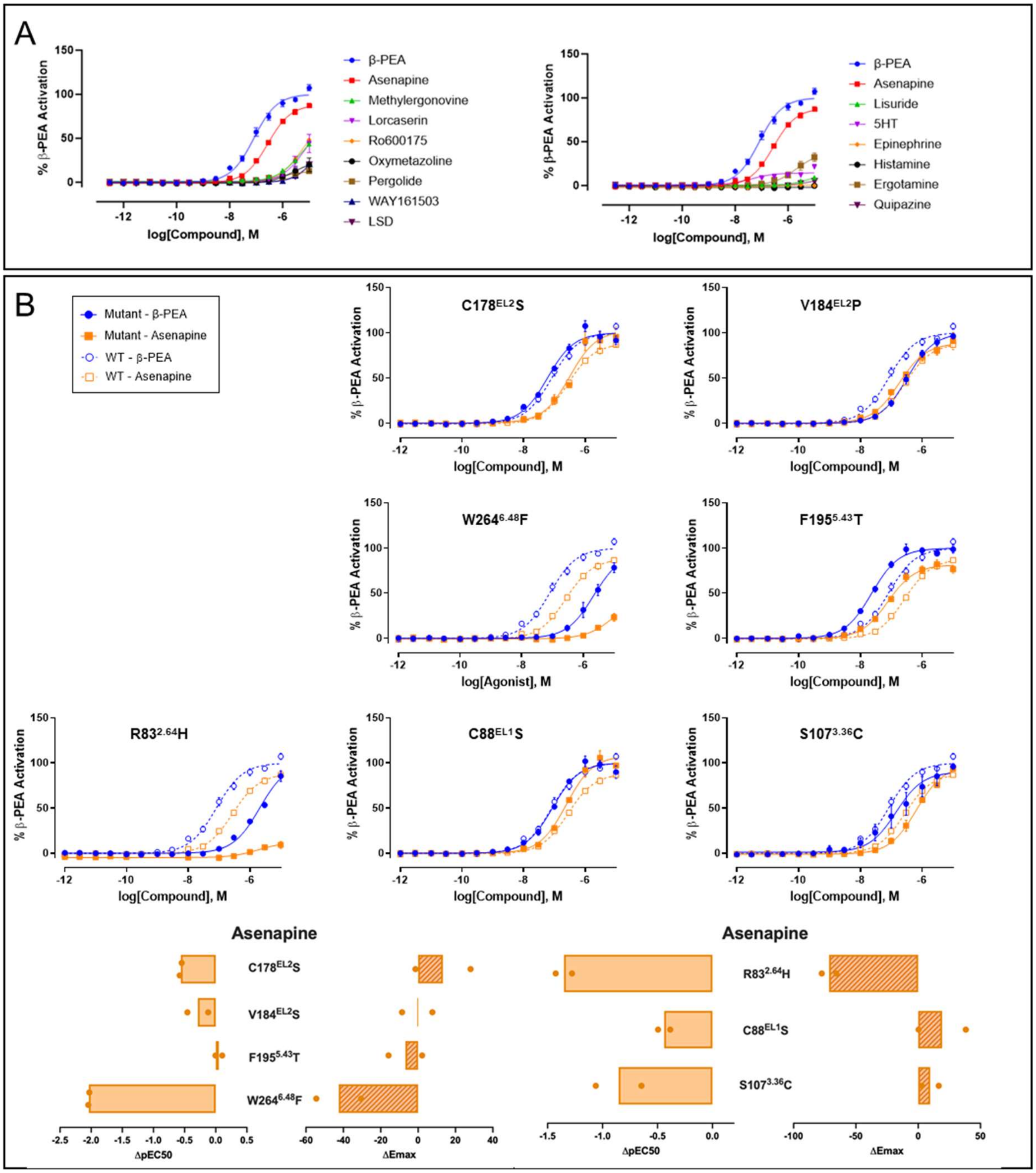

Supplementary Fig. 7 | Off-target hTA1 activities of aminergic compounds and SAR studies of asenapine at hTA1. A, Concentration response curves of top hits obtained from screening a curated aminergic compound library for hTA1 activities. Data represent mean ± SEM of two independent experiments (n=2) performed in triplicate. See Supplementary Table 3 for single concentration values of individual compounds in

screen. **B**, Effects of OBP mutations on the activity of asenapine at hTA1. Data are shown both as full concentration response curves and as differences between mutant and wild type receptor with potencies determined as pEC_{50}s and efficacies measured as E_{max}. Data represent mean ± SEM of two independent experiments (n=2) performed in triplicate. See Supplementary Table 1 for fitted parameter values.

Supplementary Table 1 | Efficacies and potencies of compounds at wildtype and mutant hTA1. ND (not determined) indicates that mutations abolish signaling. Data represent mean pEC_{50} ± SEM or mean E_{max} ± SEM normalized to β-PEA of two independent experiments (n=2) for all mutants and three independent experiments (n=3) for wt hTA1. All experiments were performed in triplicate.

	β-PEA		Tyramine		T1AM		Ro5256390		Ro5263397		Ulotaront		Ralmitaront		Asenapine	
hTA1 Construct	**EC50 (nM) (pEC50 ± SEM)**	**Span ± SEM**	**EC50 (nM) (pEC50 ± SEM)**	**Span ± SEM**	**EC50 (nM) (pEC50 ± SEM)**	**Span ± SEM**	**EC50 (nM) (pEC50 ± SEM)**	**Span ± SEM**	**EC50 (nM) (pEC50 ± SEM)**	**Span ± SEM**	**EC50 (nM) (pEC50 ± SEM)**	**Span ± SEM**	**EC50 (nM) (pEC50 ± SEM)**	**Span ± SEM**	**EC50 (nM) (pEC50 ± SEM)**	**Span ± SEM**
**WT**	80.62 (7.09 ± 0.05)	100 ± 2.26	414.87 (6.38 ± 0.04)	98.98 ± 0.26	742.61 (6.13 ± 0.04)	70.45 ± 11.17	5.33 (8.27 ± 0.08)	103.3 ± 1.72	1.47 (8.83 ± 0.09)	86.7 ± 0.98	180 (6.74 ± 0.05)	109.03 ± 5.87	110.39 (6.96 ± 0.08)	40.11 ± 14.32	273.74 (6.56 ± 0.03)	88.71 ± 2.33
**CryoEM Construct**	17.39 (7.75 ± 0.04)	100 ± 1.84	234.72 (6.62 ± 0.04)	112.93 ± 2.5	491.32 (6.3 ± 0.04)	115.44 ± 2.84	2.47 (8.6 ± 0.06)	102.23 ± 2.88	1.5 (8.82 ± 0.06)	106.6 ± 2.69	49.24 (7.3 ± 0.06)	124.95 ± 3.35	1348.3 (5.87 ± 0.04)	107.48 ± 3.32	51.76 (7.28 ± 0.04)	95.22 ± 1.98
**b2N**	25.45 (7.59 ± 0.07)	100 ± 3.11	150.67 (6.82 ± 0.05)	99.52 ± 1.00	224.09 (6.65 ± 0.05)	80.48 ± 20.30	1.56 (8.81 ± 0.07)	97.65 ± 9.34	0.66 (9.18 ± 0.09)	78.31 ± 9.88	39.64 (7.4 ± 0.06)	106.28 ± 0.92	25.69 (7.59 ± 0.06)	82.55 ± 4.39	72.78 (7.14 ± 0.06)	97.39 ± 0.96
**C5S**	90.66 (7.04 ± 0.05)	100 ± 2.22	418.22 (6.38 ± 0.05)	97.41 ± 4.98	1114.99 (5.95 ± 0.04)	81.19 ± 8.03	9.93 (8 ± 0.06)	95.38 ± 2.26	3.89 (8.41 ± 0.07)	85.5 ± 2.16	365.03 (6.44 ± 0.06)	111.01 ± 10.49	167.73 (6.78 ± 0.05)	53.02 ± 3.33	260.62 (6.58 ± 0.05)	83.24 ± 1.59
**C13S**	188.32 (6.73 ± 0.05)	100 ± 2.41	930.57 (6.03 ± 0.04)	102.3 ± 8.61	1013.87 (5.99 ± 0.05)	59.02 ± 5.94	9.21 (8.04 ± 0.06)	100.38 ± 3.73	2.48 (8.6 ± 0.08)	81.23 ± 7.68	357.44 (6.45 ± 0.04)	126.36 ± 7.99	140.12 (6.85 ± 0.1)	26.36 ± 2.60	320.93 (6.49 ± 0.05)	77.11 ± 4.84
**C88S**	84.06 (7.08 ± 0.06)	100 ± 2.93	374.73 (6.43 ± 0.04)	109.25 ± 8.40	689.69 (6.16 ± 0.04)	64.41 ± 12.82	3.1 (8.51 ± 0.04)	121.71 ± 19.43	1.64 (8.78 ± 0.06)	100.25 ± 9.11	156.05 (6.81 ± 0.04)	127.32 ± 25.39	69.97 (7.16 ± 0.05)	77.92 ± 14.98	238.04 (6.62 ± 0.04)	108 ± 18.96
**C96S**	ND	ND	ND	ND	ND	ND	ND	ND	ND	ND	ND	ND	ND	ND	ND	ND
**C178S**	59.03 (7.23 ± 0.07)	100 ± 2.97	328.08 (6.48 ± 0.04)	98.36 ± 1.77	982.78 (6.01 ± 0.05)	86.82 ± 7.63	14.14 (7.85 ± 0.05)	119.57 ± 15.22	4.26 (8.37 ± 0.09)	105.17 ± 13.16	208.98 (6.68 ± 0.06)	111.31 ± 7.17	194.78 (6.71 ± 0.04)	69.54 ± 2.90	315.45 (6.5 ± 0.07)	102.03 ± 14.74
**C88/178S**	131.00 (6.88 ± 0.05)	100 ± 2.48	516.05 (6.29 ± 0.06)	99.35 ± 4.80	1090.26 (5.96 ± 0.05)	98.9 ± 13.16	5.38 (8.27 ± 0.06)	111.12 ± 5.22	2.55 (8.59 ± 0.09)	98.55 ± 7.35	434.41 (6.36 ± 0.06)	146.47 ± 28.01	112.93 (6.95 ± 0.06)	65.82 ± 7.22	204.71 (6.69 ± 0.08)	86.32 ± 8.40
**W264F**	2494.13 (5.6 ± 0.06)	100 ± 4.75	10321.72 (4.99 ± 0.08)	100.51 ± 1.29	11327.04 (4.95 ± 0.97)	28.7 ± 10.91	126.95 (6.9 ± 0.09)	76.56 ± 14.54	1650.09 (5.78 ± 0.06)	57.37 ± 4.79	5241.11 (5.28 ± 0.06)	110.15 ± 7.15	3534.69 (5.45 ± 0.11)	40.13 ± 7.28	9426.69 (5.03 ± 0.16)	46.21 ± 12.00
**R83H**	2060.6 (5.69 ± 0.07)	100 ± 5.46	31400.86 (4.5 ± 1.31)	533.44 ± 448.15	11.69 (7.93 ± 0.33)	6.95 ± 12.95	5.19 (8.28 ± 0.11)	48.56 ± 10.14	3.18 (8.5 ± 0.15)	33.86 ± 8.09	2723.37 (5.56 ± 0.08)	102.49 ± 10.14	1.62 (8.79 ± 0.4)	2.51 ±6.63	1945.56 (5.71 ± 0.18)	17.15 ± 5.82
**D103N**	ND	ND	ND	ND	ND	ND	ND	ND	ND	ND	ND	ND	ND	ND	ND	ND
**S107C**	205.38 (6.69 ± 0.71)	100 ± 3.55	186.37 (6.73 ± 1.37)	120.36 ± 12.75	1350.62 (5.87 ± 0.53)	72.05 ± 7.70	504.39 (6.3 ± 1.55)	88.84 ± 19.77	247.82 (6.61 ± 0.80)	112.19 ± 12.31	1668.03 (5.78 ± 0.11)	197.21 ± 61.30	117.78 (6.93 ± 0.66)	49.2 ± 7.85	617.91 (6.21 ± 0.21)	98.45 ± 6.76
**V184P**	321.16 (6.49 ± 0.04)	100 ± 1.96	1490.18 (5.83 ± 0.06)	105.2 ± 5.68	539.16 (6.27 ± 0.05)	70.26 ± 20.95	3.3 (8.48 ± 0.1)	79.83 ± 1.63	1.89 (8.72 ± 0.1)	67.49 ± 4.18	143.18 (6.84 ± 0.06)	112.04 ± 2.48	272.95 (6.56 ± 0.06)	48.73 ± 0.14	167.89 (6.77 ± 0.07)	88.26 ± 8.11
**T194A**	63.62 (7.23 ± 0.13)	100 ± 2.46	26.82 (7.84 ± 0.14)	108.77 ± 4.66	235.21 (6.41 ± 0.14)	99.97 ± 36.78	34.76 (7.62 ± 0.13)	115.79 ± 9.22	56.43 (7.32 ± 0.14)	110.06 ± 28.24	1030.06 (5.86 ± 0.12)	179.74 ± 55.90	69.44 (7.33 ± 0.13)	48.89 ± 20.44	540.32 (6.34 ± 0.11)	119.91 ± 53.22
**I290N**	54.74 (7.26 ± 0.06)	100 ± 2.5	491.72 (6.31 ± 0.03)	99.42 ± 0.19	139.3 (6.86 ± 0.07)	76.97 ± 5.53	6.21 (8.21 ± 0.06)	98.98 ± 2.27	4.63 (8.33 ± 0.07)	83.69 ± 1.69	1.4 (8.85 ± 0.08)	99.97 ± 4.68	94.7 (7.02 ± 0.06)	43.63 ± 5.35	1001.59 (6 ± 0.04)	84.02 ± 6.25
**I290Y**	371.9 (6.43 ± 0.07)	100 ± 3.64	1962.68 (5.71 ± 0.07)	98.99 ± 3.20	888.62 (6.05 ± 0.08)	68.94 ± 4.39	3.79 (8.42 ± 0.08)	84.11 ± 8.72	0.69 (9.16 ± 0.18)	61.58 ± 3.99	30.31 (7.52 ± 0.11)	103.23 ± 7.35	43.71 (7.36 ± 0.17)	17.57 ± 2.61	274.98 (6.56 ± 0.08)	51.07 ± 4.43
**F195T**	23.54 (7.63 ± 0.07)	100 ±	6.34 (8.20 ± 0.59)	108.96 ± 10.72	213.01 (6.67 ± 1.32)	65.51 ± 6.79	2.90 (8.54 ± 0.14)	106.38 ± 1.33	20.93 (7.68 ± 0.60)	101.40 ± 5.29	121.61 (6.92 ± 0.11)	114.34 ± 1.09	620.51 (6.21 ± 0.79)	77.17 ± 9.48	77.08 (7.11 ± 0.05)	81.88 ± 8.97
**T271A**	701.12 (6.15 ± 0.03)	100 ± 2.03	4846.81 (5.31 ± 0.05)	123.62 ± 14.54	3515.94 (5.45 ± 0.03)	68.29 ± 26.37	14.65 (7.83 ± 0.05)	95.49 ± 3.42	5 (8.3 ± 0.07)	81.70 ± 5.69	1990.28 (5.7 ± 0.03)	124.08 ± 6.22	150.54 (6.82 ± 0.05)	61.08 ± 4.08	1152.02 (5.94 ± 0.03)	94.79 ± 5.91
**T271N**	374.35 (6.43 ± 0.06)	100 ± 3.3	862.88 (6.06 ± 0.04)	102.55 ± 3.93	560.2 (6.25 ± 0.03)	119.8 ± 27.24	12.58 (7.9 ± 0.07)	110.48 ± 10.78	5 (8.3 ± 0.07)	108.08 ± 8.54	1408.35 (5.85 ± 0.03)	133.69 ± 19.67	118.86 (6.92 ± 0.05)	87.44 ± 33.75	478.81 (6.32 ± 0.04)	121.93 ± 21.07

Supplementary Table 2 I Cryo-EM data collection, refinement and validation statistics.

	**Ro5256390/ hTA1-Gs-Nb35** (EMD-42268) (PDB ID: 8UHB)
**Data collection and processing**	
Magnification	64,000
Voltage (kV)	300
Electron exposure (e–/Å^2)	53.88
Defocus range (μm)	-0.5 to -1.8
Pixel size (Å)	1.069
Symmetry imposed	C1
Initial particle images (no.)	6,413,421
Final particle images (no.)	626,730
Map resolution (Å)	3.35
FSC threshold	0.143
Map sharpening B-factor (Å^2)	-160.3
**Refinement**	
Model composition	
Non-hydrogen atoms	8417
Protein residues	1075
Ligands	1
R.m.s. deviations	
Bond lengths (Å)	0.004
Bond angles (°)	0.619
Validation	
Clashscore	9.16
Poor rotamers (%)	0
Ramachandran plot	
Favored (%)	94.15
Allowed (%)	5.85
Disallowed (%)	0

Supplementary Table 3 | Activity of 10 µM aminergic drugs at hTA1 as determined by cAMP accumulation in HEK293T cells. Experiment was performed in quadruplicate. Compounds that met the activation threshold of 2*log2 fold change (log2fc) over DMSO baseline are highlighted in green.

Compound	Log2fc of DMSO ± SD
2-Bromo-LSD	0.204 ± 0.413
5-Methoxytryptamine	0.523 ± 0.199
5-HT	1.794 ± 0.316
Alprenolol	0.287 ± 0.164
Altanserin	0.684 ± 0.246
Amisulpride	0.706 ± 0.297
Amphetamine	4.026 ± 0.304
Aripiprazole	0.535 ± 0.101
AS19	0.363 ± 0.116
Asenapine	5.497 ± 0.09
βPEA	4.639 ± 0.205
BRL54443	0.531 ± 0.155
Buspirone	0.318 ± 0.156
BW723C86	1.446 ± 0.158
Cabergoline	0.71 ± 0.188
Carvedilol	-0.068 ± 0.072
Chlorpromazine	-0.609 ± 0.29
Clozapine	0.49 ± 0.077
CNO	0.716 ± 0.038
Cyanopindolol	0.302 ± 0.282
Cyproheptadine	0.016 ± 0.047
Dihydroergotamine	-0.486 ± 0.177
DMSO	0.025 ± 0.571
Donitriptan	1.034 ± 0.065
DR 4485	-0.406 ± 0.338
Efavirenz	-0.465 ± 0.094
Epinephrine	2.118 ± 0.31
Ergotamine	2.943 ± 0.086
Fananserin	0.199 ± 0.111
Fluoxetine	0.566 ± 0.062
Flupentixol	0.441 ± 0.111
Fluphenazine	0.327 ± 0.238
GBR12909	-1.05 ± 0.147
GMD281014	-0.399 ± 0.181
Haloperidol	0.713 ± 0.2
Histamine	2.117 ± 0.232
Ipsapirone	-0.011 ± 0.211
Ketanserin	-0.697 ± 0.174
LE300	0.473 ± 0.259
Lisuride	3.176 ± 0.306
Lobeline	0.044 ± 0.12
Lorcaserin	4.221 ± 0.105
LP12	0.652 ± 0.109
LSD	2.923 ± 0.238

Compound	Log2fc of DMSO ± SD
LuAE58085	-0.537 ± 0.285
Lurasidone	0.764 ± 0.17
LY266097	0.745 ± 0.161
LY393558	-0.068 ± 0.169
MDL109	0.532 ± 0.138
Mesulergine	0.848 ± 0.156
Methiothepin	0.061 ± 0.516
Methylergonovine	3.724 ± 0.197
NAD299	0.499 ± 0.12
NAN190	0.472 ± 0.045
Nemonapride	0.693 ± 0.106
NPS ALX Compound 4a	0.113 ± 0.157
Olanzapine	0.461 ± 0.114
Oxymetazoline	2.307 ± 0.106
Paliperidone	0.617 ± 0.048
Paroxetine	0.691 ± 0.092
Pergolide	2.403 ± 0.197
Pimethixene	-0.362 ± 0.331
Pimozide	0.756 ± 0.097
Prazosin	-0.306 ± 0.266
Propanolol	0.404 ± 0.219
Quinpirole	1.96 ± 0.167
Quipazine	2.286 ± 0.178
Raclopride	-0.389 ± 0.224
Rauwolscine	0.086 ± 0.118
Reserpine	0.436 ± 0.395
Risperidone	0.531 ± 0.099
Ritanserin	0.538 ± 0.204
Ro600175	3.659 ± 0.062
RS 39604	-0.323 ± 0.189
RS 127445	1.34 ± 0.122
SB204741	-0.001 ± 0.155
SB269970	0.493 ± 0.105
SB399885	-0.563 ± 0.171
SCH 23390	0.589 ± 0.103
Sertindole	1.07 ± 0.185
Spiperone	0.261 ± 0.144
Sumatriptan	0.746 ± 0.109
Tetrabenazine	0.51 ± 0.217
Terguride	0.783 ± 0.353
WAY161503	2.401 ± 0.253
Yohimbine	-0.506 ± 0.145
Ziprasidone	0.339 ± 0.302
Zolmitriptan	0.583 ± 0.146
Zotepine	0.272 ± 0.11

Supplementary Data

An uncropped gel of the gel provided in Supplementary Figure 1. Relevant lanes are indicated by a box, all other lanes correspond to material unrelated to this study.

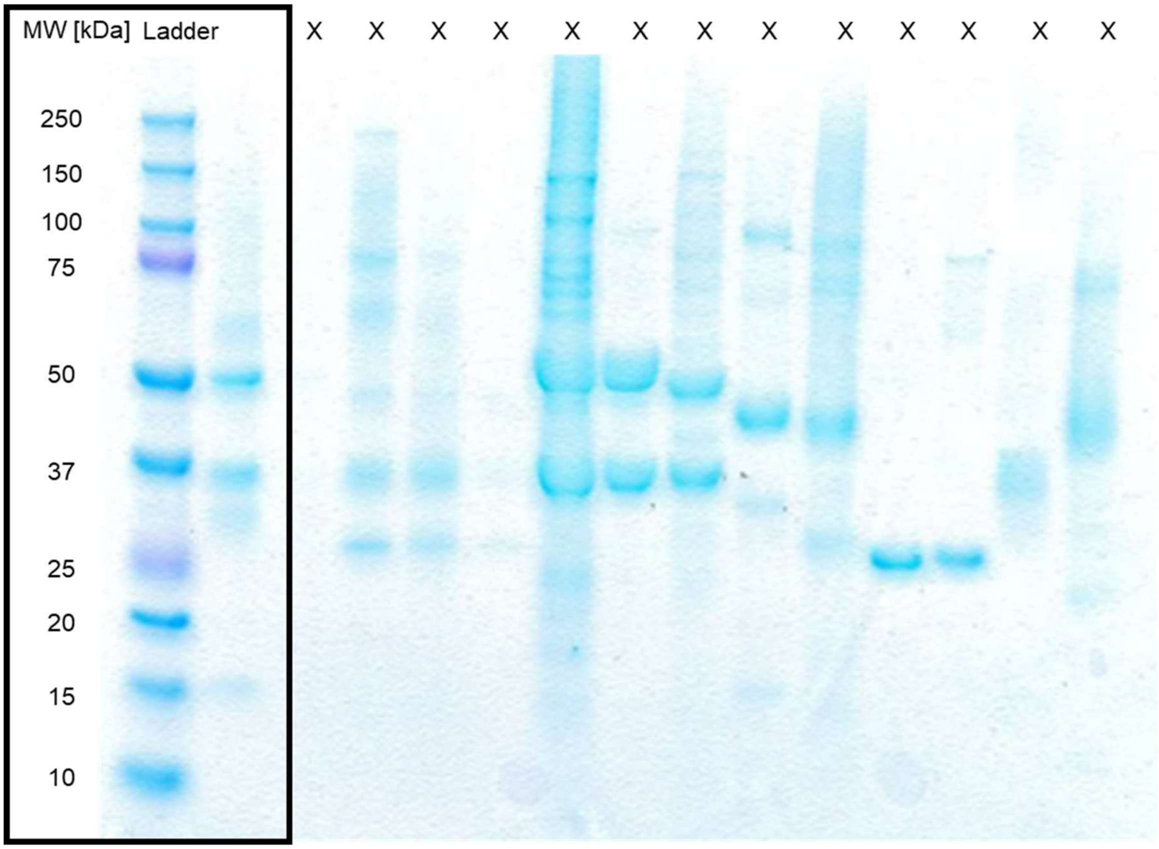

Supplementary Primer Information

Oligonucleotides used to generate the mutants characterized in this publication.

TA1 I290Y F	GACGTGCTTTACTGGTTCGGCTAT
TA1 I290N F	GACGTGCTTAACTGGTTCGGCTAT
TA1 I290mut R	GTTCAGGGTGGGCGGTAT
TA1 F112W F	CGCTTCAATCTGGCACCT
TA1 F112W R	GGTGCCAGATTGAAGCGCTGC
TA1 C5S F	CTTTTTCTCATAATATC
TA1 C5S R	ATGAGAAAAAGGCATCATATC
TA1 C13S F	CAACATTTCCTCCGTG
TA1 C13S R	CAATTGTTTTTCACGGAGGAAATG
TA1 C88S F	CTCTGCCGAGCACTCTTGG
TA1 C88S R	CGAAATACCAAGAGTGC
TA1 C96S F	CGAAGTGTTCTCCAAAATTC
TA1 C96S R	GAATTTTGGAGAACAC

TA1 C178S F	CATGTCCACTCTCGG
TA1 C178S R	CCACCCCGAGAGTGG
TA1 C182S F	CGGGGTGGATCCTCCG
TA1 C182S R	CTAAAAAACACGGAGGATCCACC
TA1 D103N F	GTCTACGAATATCATGCTCAGC
TA1 D103N R	CATGATATTCGTAGACGTGTGAATTTTG
TA1 F195T F	GTCCTGACGACCATGACTTCCT
TA1 F195T R	GTCATGGTCGTCAGGACGC
TA1 R83H F	GGTCCACTCTGCCGAGCAC
TA1 R83H R	GGCAGAGTGGACCATGCTGTAG
TA1 V184P F	ATGCTCCCCGTTTTTTAG
TA1 V184P R	AAAACGGGGAGCATCC
TA1 T194A F	TCCTGGCGTTCATGAC
TA1 T194A R	AACGCCAGGACGC
TA1 W264F F	CTGATATGTTTTTGTCCTTTCTTC
TA1 W264F R	AGGACAAAAACATATCAGAAAGAC
TA1 T271N F	CATTTGTAATGTCATGGACCC
TA1 T271N R	TGACATTACAAATGAAGAAAGGAC
TA1 T271A F	TTTGTGCTGTCATGGAC
TA1 T271A R	ACAGCACAAATGAAGAAAG
TA1 S107C F	TCTGCAGCGCTTCAATC
TA1 S107C R	GCTGCAGAGCATGATATCC
