# Supplementary material for: Molecular basis of human trace amine-associated receptor 1 activation
Source: Nat Commun. 2024 Jan 2;15:108. doi: 10.1038/s41467-023-44601-4 (PMC10762035; doi:10.1038/s41467-023-44601-4)
Supplement: Supplementary file 3 — Description of Additional Supplementary Files [file 41467_2023_44601_MOESM3_ESM.pdf]

Description of Additional Supplementary Files

File Name: Supplementary Movie 1
Description: 3D variability analysis of the Ro5256390-bound hTA1-Gs-Nb35 complex reveals flexibility at receptor the N-terminus.

File Name: Supplementary Movie 2
Description: 3D variability analysis of the Ro5256390-bound hTA1-Gs-Nb35 complex reveals movements of receptor TM1.

File Name: Supplementary Movie 3
Description: 3D variability analysis of the Ro5256390-bound hTA1-Gs-Nb35 complex reveals movements of receptor TM4.

File Name: Supplementary Movie 4
Description: 3D variability analysis of the Ro5256390-bound hTA1-Gs-Nb35 complex reveals flexibility at the intracellular tips of TM5 and TM6.

File Name: Supplementary Movie 5
Description: 3D variability analysis of the Ro5256390-bound hTA1-Gs-Nb35 complex reveals twisting motion of the receptor TM bundle.
