# Supplementary material for: Molecular basis of human trace amine-associated receptor 1 activation
Source: Nat Commun. 2024 Jan 2;15:108. doi: 10.1038/s41467-023-44601-4 (PMC10762035; doi:10.1038/s41467-023-44601-4)
Supplement: Supplementary file 9 — Reporting Summary [file 41467_2023_44601_MOESM9_ESM.pdf]

nature portfolio

Corresponding author(s): Daniel Wacker

Last updated by author(s): Dec 14, 2023

Reporting Summary

Nature Portfolio wishes to improve the reproducibility of the work that we publish. This form provides structure for consistency and transparency in reporting. For further information on Nature Portfolio policies, see our Editorial Policies and the Editorial Policy Checklist.

Statistics

For all statistical analyses, confirm that the following items are present in the figure legend, table legend, main text, or Methods section.

n/a	Confirmed	
☐	☒	The exact sample size (*n*) for each experimental group/condition, given as a discrete number and unit of measurement
☐	☒	A statement on whether measurements were taken from distinct samples or whether the same sample was measured repeatedly
☒	☐	The statistical test(s) used AND whether they are one- or two-sided<br>*Only common tests should be described solely by name; describe more complex techniques in the Methods section.*
☒	☐	A description of all covariates tested
☒	☐	A description of any assumptions or corrections, such as tests of normality and adjustment for multiple comparisons
☐	☒	A full description of the statistical parameters including central tendency (e.g. means) or other basic estimates (e.g. regression coefficient) AND variation (e.g. standard deviation) or associated estimates of uncertainty (e.g. confidence intervals)
☒	☐	For null hypothesis testing, the test statistic (e.g. *F*, *t*, *r*) with confidence intervals, effect sizes, degrees of freedom and *P* value noted<br>*Give P values as exact values whenever suitable.*
☒	☐	For Bayesian analysis, information on the choice of priors and Markov chain Monte Carlo settings
☒	☐	For hierarchical and complex designs, identification of the appropriate level for tests and full reporting of outcomes
☒	☐	Estimates of effect sizes (e.g. Cohen's *d*, Pearson's *r*), indicating how they were calculated

Our web collection on statistics for biologists contains articles on many of the points above.

Software and code

Policy information about availability of computer code

Data collection	CryoEM data collection on Titan Krios electron microscope equipped with a K3 direct electron detector
Data analysis	CryoSPARC v4.1.2., MotionCor2, Ctffind4, WinCoot v0.9.8.1, Phenix v1.19.2, MolProbity, PyMol v2.5.4, UCSF Chimera v1.16, UCSF ChimeraX v1.2.5, GraphPad Prism8, ServalCat (embedded in CCPEM 1.6.0)

For manuscripts utilizing custom algorithms or software that are central to the research but not yet described in published literature, software must be made available to editors and reviewers. We strongly encourage code deposition in a community repository (e.g. GitHub). See the Nature Portfolio guidelines for submitting code & software for further information.

Data

Policy information about availability of data

All manuscripts must include a data availability statement. This statement should provide the following information, where applicable:

- Accession codes, unique identifiers, or web links for publicly available datasets
- A description of any restrictions on data availability
- For clinical datasets or third party data, please ensure that the statement adheres to our policy

Density maps and structure coordinates have been deposited in the Electron Microscopy Data Bank (EMDB- 42268) and the PDB (PDB ID: 8UHB), and the micrographs have been deposited to EMPIAR (EMPIAR-11755)

Research involving human participants, their data, or biological material

Policy information about studies with human participants or human data. See also policy information about sex, gender (identity/presentation), and sexual orientation and race, ethnicity and racism.

Reporting on sex and gender	N/A
Reporting on race, ethnicity, or other socially relevant groupings	N/A
Population characteristics	N/A
Recruitment	N/A
Ethics oversight	N/A

Note that full information on the approval of the study protocol must also be provided in the manuscript.

Field-specific reporting

Please select the one below that is the best fit for your research. If you are not sure, read the appropriate sections before making your selection.

☒ Life sciences ☐ Behavioural & social sciences ☐ Ecological, evolutionary & environmental sciences

For a reference copy of the document with all sections, see nature.com/documents/nr-reporting-summary-flat.pdf

Life sciences study design

All studies must disclose on these points even when the disclosure is negative.

Sample size	Sample size was not predetermined. Sample sizes were chosen based on years of experience with in vitro signaling aassays, as well as based on sample sizes generally used and accepted in the field. We generally used sample sizes of 2 or 3 independent experiments done with 3 or more technical repeats to show reproducibility.
Data exclusions	No data was excluded
Replication	Experimental findings were reproduced, and number of technical repeats and independent experiments has been provided in the methods and figure legends.
Randomization	In preliminary experiments we observe low variability in the expression of receptor constructs between cells cultured in neighboring wells of 384-well plates. Based on these results we did not perform randomization.
Blinding	No blinding was required for the in vitro experiments performed in this study, as all measures rely on instrument-based quantification of fluorescence and no subjective assessment of data that influences the outcome of the analysis.

Reporting for specific materials, systems and methods

We require information from authors about some types of materials, experimental systems and methods used in many studies. Here, indicate whether each material, system or method listed is relevant to your study. If you are not sure if a list item applies to your research, read the appropriate section before selecting a response.

Materials & experimental systems

n/a	Involved in the study	
☐	☒	Antibodies
☐	☒	Eukaryotic cell lines
☒	☐	Palaeontology and archaeology
☒	☐	Animals and other organisms
☒	☐	Clinical data
☒	☐	Dual use research of concern
☒	☐	Plants

Methods

n/a	Involved in the study	
☒	☐	ChIP-seq
☒	☐	Flow cytometry
☒	☐	MRI-based neuroimaging

Antibodies

Antibodies used	gp64-PE antibody, Expression Systems, Cat No: 97-201, Clone: AcV1, Lot No: 22102021
Validation	No validation of the antibody was performed

Eukaryotic cell lines

Policy information about cell lines and Sex and Gender in Research

Cell line source(s)	Sf9 cells (Expression Systems), HEK293-T Cell Line (ATCC)
Authentication	None of the cell lines used was authenticated
Mycoplasma contamination	All cell lines used in this study tested negative for mycoplasma contamination
Commonly misidentified lines (See ICLAC register)	No commonly misidentified cell lines were used
